# Relating mineral–organic matter stabilization mechanisms to carbon quality and age distributions using ramped thermal analysis

**DOI:** 10.1098/rsta.2023.0139

**Published:** 2023-11-27

**Authors:** Shane Stoner, Susan E. Trumbore, José A. González-Pérez, Marion Schrumpf, Carlos A. Sierra, Alison M. Hoyt, Oliver Chadwick, Sebastian Doetterl

**Affiliations:** ^1^ Department of Biogeochemical Processes, Max Planck Institute for Biogeochemistry, Jena, Germany; ^2^ Department of Environmental Systems Science, ETH Zürich,8092 Zurich, Switzerland; ^3^ Biogeoquímica, Ecología Vegetal y Microbiana, Instituto de Recursos Naturales y Agrobiología de Sevilla, CSIC, Sevilla, Spain; ^4^ Earth System Science, Stanford University, Stanford, CA 94305, USA; ^5^ Department of Geography, University of California, Santa Barbara, CA, USA

**Keywords:** radiocarbon, soil organic matter, mineral-associated organic matter, py-GC/MS, soil minerals

## Abstract

Organic carbon (OC) association with soil minerals stabilizes OC on timescales reflecting the strength of mineral–C interactions. We applied ramped thermal oxidation to subsoil B horizons with different mineral–C associations to separate OC according to increasing temperature of oxidation, i.e. thermal activation energy. Generally, OC released at lower temperatures was richer in bioavailable forms like polysaccharides, while OC released at higher temperatures was more aromatic. Organic carbon associated with pedogenic oxides was released at lower temperatures and had a narrow range of ^14^C content. By contrast, N-rich compounds were released at higher temperatures from samples with 2 : 1 clays and short-range ordered (SRO) amorphous minerals. Temperatures of release overlapped for SRO minerals and crystalline oxides, although the mean age of OC released was older for the SRO. In soils with more mixed mineralogy, the added presence of older OC released at temperatures greater than 450°C from clays resulted in a broader distribution of OC ages within the sample, especially for soils rich in 2 : 1 layer expandable clays such as smectite. While pedogenic setting affects mineral stability and absolute OC age, mineralogy controls the structure of OC age distribution within a sample, which may provide insight into model structures and OC dynamics under changing conditions.

This article is part of the Theo Murphy meeting issue ‘Radiocarbon in the Anthropocene’.

## 1. Introduction

Soils hold the largest terrestrial reservoir of organic carbon (OC) (1500 petagrams in the top 100 cm, [1]), and understanding the persistence of OC in soil is key to predicting soil feedbacks to changing climate. Importantly, the role of soil minerals has been established as a key mechanism for stabilizing soil organic matter (SOM) [2–4] in both top and subsoils. However, studies on SOM stabilization in the subsoil are more limited [5,6] despite the predominance of mineral–OM interactions [3,7], less saturation of mineral surfaces [8,9] and evidence of the reduced influence of surface plant-derived C [10,11].

The reactive mechanisms linking mineral surfaces and SOM are diverse and include processes such as sorption, complexation, ligand exchange and chelation, as well as stronger interactions with minerals having permanent surface charge such as 2 : 1 clays [3,4]. The strength of these interactions helps to determine how long OC persists. Total SOM storage has been linked to (pedogenic) Fe and Al oxy-(hydr)oxides concentrations [12,13], even in mixed mineralogy and clay-rich soils [14–17]. Soils rich in reactive short-range order (SRO) non-crystalline Al, Fe and Mn oxides strongly complex large quantities of SOM via dissociated functional groups, forming strong inner-sphere bonds [2,18,19]. Further weathering of these Fe and Al oxy-(hydr)oxides to secondary minerals produces lower-entropy crystalline oxides (CO), such as haematite and goethite, that are significantly less reactive and store less SOM.

SOM associations with silicate layer clay minerals depend strongly on the forms of clay present. For example, high-surface area 2 : 1 clays can adsorb large molecules through cation bridging (e.g. Ca^2+^) between negative charges on clay surfaces and negatively charged SOM functional groups (particularly carboxyl groups) [20]. Aromatic SOM can form hydrophobic microsites which may hinder decomposition and stimulate further accumulation of SOM through OM–OM interactions [21]. In addition, positively charged edges of 2 : 1 clay minerals may directly complex OM through rapid ligand exchange [22,23]. 1 : 1 layer silicate clays have much lower surface area and primarily form complexes at edge sites [24]. They can, however, be coated with more reactive Fe and Al oxides, as is typically observed in Oxisols [25,26]. Thus, multiple stabilization pathways can exist on and between individual minerals, e.g. edge charges and isomorphic substitutions in 2 : 1 clays, leading to a distribution of bond strengths and SOM resiliency [4].

Reactive soil mineral surfaces and OC input to soil are spatially heterogeneous. Notably, physical occlusion mechanisms can play a role in long-term persistence by isolating SOM from decomposers and extracellular enzymes [27,28]. Stable microaggregates formed by a mix of pedogenic (oxy)hydroxides, clay minerals and SOM present an efficient barrier for many microorganisms leading to the preservation of otherwise readily available SOM. Thus, the age of OC associated with the same minerals may differ depending on the stability of the structure in which these minerals are embedded.

In addition to mineral stabilization, the persistence of OM in soil has been attributed to chemical ‘recalcitrance’, or the difficulty with which soil microbes break bonds within an organic molecule, e.g. differentiating low molecular weight fresh sugars from more stable biopolymers such as lignin or poorly recognizable charred aromatic OM. The influence of chemical complexity along with the potential for one or more mineral stabilization mechanisms to select for organic molecules with specific properties likely results in quantifiable differences in the distribution of activation energies that control the time until a given organic molecule is made available for decomposition and its C released from soil back to the atmosphere [29,30].

The OC in SOM is known to have a range of ages that are related to soil mineralogy. Recent advances in multi-pool SOM models allow the calculation of the probabilistic ^14^C distribution for bulk SOM sampled at a given point in time [31]. This provides a method for comparison of models with ^14^C observations directly against either time series of samples from the same site or the age distribution of a single sample determined using laboratory physical or chemical fractionation. Thermal fractionation, which measures SOM decomposition into CO_2_ under increasing energy inputs (i.e. heat) can now be used to isolate the ^14^C signal of quantitatively and qualitatively distinct parts of SOM that differ in thermal stability, molecular structure and bonding mechanisms [32,33]. By using the temperature of thermal decomposition as a proxy for activation energy, the ^14^C distributions of OC can be compared with other fractionation approaches [34], time series [35] or model-predicted distributions [31]. Thus, thermal analysis results do not reflect just resistance of organic molecules to withstand thermally induced decomposition, but aggregate the strength of mineral sorption and complexes alongside thermal stability and chemical complexity. For example, Feng & Simpson [36] showed that the same molecule can have different thermal activation energies depending on the associated mineral matrix.

Given the wide range of potential physical and chemical interactions, SOM associated with various soil minerals differs greatly in quality, quantity and mean age, represented by the time OC has persisted in the soil. In order to predict SOM dynamics and parameterize SOM turnover in models, it is crucial to accurately represent different timescales of SOM stabilization and their patterns across soil types. Most models of SOM dynamics [37–39] require at least two ‘pools’ of SOM with different characteristic rates of decomposition to represent changes in SOM dynamics following e.g. land use change [40–43]. These decomposition rates are often linked to characteristics like soil clay content that provide a proxy for different timescales of mineral–OC interactions.

Experimentally, ^14^C analyses provide one of the only tools to quantify the age and rate of cycling of soil OC. However, relatively few studies have linked ^14^C to mineral stabilization mechanisms directly [17,44,45]. While ^14^C measurements of bulk SOM are increasingly common, mean values insufficiently describe SOM dynamics. This is evidenced by the rapid incorporation of recent bomb ^14^C into SOM with mean ^14^C ages of hundreds to thousands of years, or the ability of various chemical and physical fractionation methods to separate bulk SOM into fractions with different mean ages (e.g. [46–49].

In this study, we explore the potential of thermal analysis to improve the description of ^14^C-derived age distributions of mineral-associated organic matter (MOM) for geochemically and pedogenetically distinct soils with a wide range of mineral stabilization mechanisms. To address this, we proposed the following questions and hypotheses:

1. *How does mineralogy control MOM age structure and thermal stability?* We hypothesize that variation in bonding mechanisms between SOM and minerals in different soils corresponds to variation in activation energy required to mineralize SOM and this will be reflected in the mean age of the OC oxidized at different temperatures. We further predict that the oldest OC would be associated with minerals with the strongest stabilization pathways—i.e. soils rich in amorphous short-range ordered (SRO) minerals require the highest amount of activation energy (most stable organo-mineral bonds and older C) followed by soils rich in expandable 2 : 1 clay minerals and pedogenic oxides. Mineralizing SOM in soils rich in end-member minerals such as non-expandable 1 : 1 clays, quartz and highly crystalline pedogenic oxides should require only a low amount of activation energy (least stable organo-mineral bonds and youngest C). Similarly, soils rich in primary minerals where soil development has not yet created strong organo-mineral bonds with secondary minerals will show comparatively low activation energy and younger C.
2. *How does mineralogy control MOM chemistry?* We hypothesize that the relationship between thermal stability and mean age is driven primarily by the ability of soil minerals to stabilize organic molecules, and only secondarily by their biochemical molecular structure. As such, soils with the strongest mineral-related stabilization mechanisms will also have the ability to stabilize potentially bioavailable organic molecules, whereas soils with weak mineral-related stabilization mechanisms can be dominated by molecules that show inherent resistance to decomposition.

To test these hypotheses, we selected mineral fractions from soil B horizons, where mineral–organic interactions should dominate SOM dynamics. We chose soil B horizons because mineral–OM interactions likely dominate there, and because of recent studies suggesting that a portion of B horizon OM is cycling on timescales of decades [50] and is vulnerable to destabilization with soil warming [51]. To maximize the variation of expected mineral–organic matter interactions, we selected soils with previously quantified mineralogy that developed in different soil environments on a range of parent materials. These samples included previously investigated soils from a chronosequence study [52], and soils with similar age and climate but different parent materials [16,53], as well as soils with unique mineral–OM stabilization mechanisms, e.g. a Spodosol [54,55]. We subjected these samples to thermal fractionation, trapping the CO_2_ oxidized in different temperature ranges to determine patterns in quantity, ^14^C, and ^13^C of released C. The same samples also underwent Rock-Eval pyrolysis, an assessment of SOM evolution and maturity, and pyrolysis gas chromatography and mass spectrometry (py-GC/MS) to characterize the chemical composition of the MOM in each soil. By linking chemistry and ^14^C thermal analysis to distinct thermal fractions of MOM, we provide new insights on characterizing MOM composition and move beyond bulk ^14^C ages to better link soil minerals to the age structure of SOM.

## 2. Methods

### (a) Sample selection and site description

Samples were selected from well-characterized B horizons of soils representing a variety of mineralogies, including those with single mineral classes and combined mineralogies to test endpoints and mixtures (table 1). The influence of fresh plant inputs was minimized by removing free particulate organic matter (FPOM) using density separation and analysing only MOM. No soils contained carbonates, ensuring that all C released during thermal oxidation is derived from MOM. For simplicity, each sample is named to express its dominant mineral(s). Soil characteristics and relevant citations are presented in table 1. For example, ‘Quartz’ is from the quartz-rich Bh horizon of a Podzol developed on aeolian dune sands since the last ice age [54]. All other soils analysed were developed on igneous parent materials of varying geochemical composition to exclude ^14^C-free OC inherited from sedimentary mineral–OC associations. A previous publication demonstrated the potential for inherited OC from sedimentary parent material to influence the thermal analysis [34,59].

**Table 1.** Properties of selected soils, in order of lowest to highest degree of weathering. B horizons were selected to isolate unsaturated mineral phases. ‘Fe dith’ is dithionite extractable iron, and ‘Fe ox’ and‘Al ox’ are oxalate extractable iron and aluminium, respectively.

			depth of B	MOM Fm	MOM Fm	depth-corrected	MOM													
group	sample ID	location	horizon	^14^C	age^a^	Fm^b^	*δ*^13^C	organic C	pH	Fe dith	Fe ox	Al ox	clay	kaolin	smectite	chlorite	feldspar	quartz	GPP^c^	citation
			cm		year BP	45 cm	‰	%		g kg^−1^	g kg^−1^	g kg^−1^	%	% of clay-sized fraction^d^					g C m^−2^ y^−1^	
quartz sand dominated	Quartz	Michigan, USA	56–76	0.907	800	0.90	−25.32	1.0	4.0	1.50	0.18	0	0	0	0	0	5	89	1589	[54,55]
primary mineral dominated	Int. PM + SRO	Sierra Nevada Range, USA	20–30	0.892	900	0.88	−23.21	1.1	6.0	16.03	6.92	38.58	6	6	36	2	31	2	1508	[17,53]
primary mineral dominated	Felsic PM + Mixed Clay	Sierra Nevada Range, USA	20–30	0.987	100	0.91	−23.75	0.4	6.1	4.30	2.60	4.4	6	3	1	5	40	23	1279	[17,53]
primary mineral dominated	Mafic PM + CO	Sierra Nevada Range, USA	20–30	0.938	500	0.87	−22.01	0.8	6.0	9.57	3.47	14.75	11	12	0	0	41	0	1431	[17,53]
amorphous mineral dominated	SRO	Hawaii, USA	38–54	0.674	3200	0.68	−26.69	5.9	4.8	24.20	8.82	29.4	4	1	0	0	2	6	2315	[2,10,52]
clay dominated	2 : 1 Clay + CO	South Africa	11–31	0.873	1100	0.79	−12.3	1.3	7.7	2.90	0.12	2	49	5	93	0	0	2	1088	[16]
clay dominated	Mixed Clay + Quartz	South Africa	15–29	1.009	Modern	1.00	−13.8	0.5	6.8	0.16	0.07	0.7	47	53	24	15	0	44	1528	[16]
clay dominated	1 : 1 Clay + Quartz	South Africa	41–62	0.921	650	0.94	−14.9	0.2	5.6	0.36	0.03	0	17	79	1	21	0	75	1528	[16]

^a^MOM age calculated using a one-pool model as described by Khomo *et al*. [56].

^b^Estimated ^14^C at 45 cm depth calculated via mass-preserving quadratic spline method [57] using R package ‘mpspline2’ (see electronic supplementary material, figure S1).

^c^Data from Zhang *et al*. [58].

^d^Data for soil ‘SRO’ and ‘CO + 1 : 1 Clay’ from less than 2 mm fraction.

#### (i) Amorphous mineral-dominated soils

We selected a volcanic soil rich in amorphous minerals, with a high OC content. Site ‘SRO’ is dominated by reactive secondary SRO minerals. The ‘SRO’ soil was collected from site Laupahoehoe of the LSAG parent material weathering chronosequence in Hawaii (montane rainforests, MAT = 16°C, MAP = ∼2500 mm, parent material mixture of volcanic tephra and lava) [2,52,60]. ‘SRO’ is classified as an Andisol (Aquic Hydrudand) developed on a 20 000-year-old lava flow. The soil is composed of the weathering products of olivine, pyroxene and plagioclase that are dominated by amorphous meta-stable SRO minerals like ferrihydrite, nanogoethite and poorly crystalline forms of oxides and aluminosilicates including allophane [2].

#### (ii) Clay-dominated soils

Soils that represent an array of clay mineral compositions were selected from previous studies in Kruger National Park in South Africa [16,56,61,62], from sites with similar climate conditions and savannah vegetation. These soils have very low SRO or primary mineral content, and the main stabilization mechanisms are associated with crystalline clay minerals (table 1). Sites ‘1 : 1 Clay + Quartz’ and ‘Mixed Clay + Quartz’ were collected from a catena toposequence developed on granitic parent material and represent the crest and toeslope positions, respectively, with a midslope seep and leaching zone in between. ‘1 : 1 Clay + Quartz’ (crest position) is classified as a lower productivity Entisol (Ustorthent) with a clay fraction (17% texture) dominated by non-expandable 1 : 1 clays with some non-expandable 2 : 1 chlorite (table 1). ‘Mixed Clay + Quartz’ (toeslope position) is classified as a higher productivity Alfisol (Natrusalf) with higher clay content (47% texture) and higher amounts of expandable 2 : 1 clays including smectite. ‘2 : 1 Clay + CO’ is classified as a Vertisol (Haplustert), developed on basalt parent material. It is a high-clay (43% texture), low-oxide soil with relatively high SOM stocks. It is dominated by smectite (93% of the clay-sized fraction), thus representing an expandable 2 : 1 clay-dominated soil.

#### (iii) Primary mineral-dominated soils

To contrast soils rich in secondary minerals with those in early development stages, we included soils with varying parent material from a cooler climate transect site in the Sierra Nevada mountains in California, USA [17,53]. These samples have significant concentrations of the primary mineral feldspar and a large variation in mineral reactivity due to differences in the geochemistry of soil parent material. ‘Felsic PM + Mixed Clay’ is classified as an Inceptisol (Dystroxerept) developed on granite parent material and contains low levels of SRO and clay minerals. ‘Int. PM + SRO’ is classified as an Andisol (Haploxerand) developed on andesite, an intermediate igneous parent material. It contains high levels of SRO minerals and a small amount of smectite. ‘Mafic PM + CO’ is classified as an Inceptisol (Haploxerept) developed on mafic basalt parent material containing intermediate levels of SRO and comparably low clay content (11% by texture).

#### (iv) Quartz sand-dominated soils

As a measure of OC stabilization and turnover in soils nearly devoid of reactive minerals (including clays), we selected soil developed on post-glacial dune sands located in northern Michigan, USA. ‘Quartz’ is classified as a Spodosol (Entic Haplorthod) where the only mineral-derived stabilization mechanism is (oxy)hydroxides precipitated on sand particles. Although classified as a Spodosol, the concentrations of extractable metals from this mineral B horizon are low (table 1, [55]).

### (b) Thermal analysis

#### (i) Thermal fractionation

For our analysis, we selected only the MOM fraction in the B horizons of the investigated soils after density separation (1.9 g cm^−2^ sodium polytungstate (SPT) solution) for thermal analysis to remove low-density particulate OM. Note that samples ‘1 : 1 Clay + Quartz’, ‘2 : 1 Clay + CO’ and ‘Mixed Clay + Quartz’ were not density fractionated, but were determined to have low (less than 5% of soil OC) FPOM concentrations in prior work [16]. Furthermore, since substantial amounts of OC were lost from ‘Quartz’ via dissolution in SPT an unfractionated bulk sample was also analysed for comparison (electronic supplementary material, figures S2–S5).

Methods for thermal fractionation are described in detail by Stoner *et al*. [34] and elsewhere [32,33]. First, temperature ranges of CO_2_ collection (approx. 200 to approx. 500°C) are selected by producing an initial profile of OC release (thermogram) which is deconvolved and transformed to a probability distribution of activation energy (*E*_a_) based on the time-temperature relationship of sample collection (below, [33]). Component peaks underlying the thermogram can then be represented as Gaussian distributions, and thermal ‘fractions’ of OC can be isolated by trapping CO_2_ released in specific temperature ranges(s). Using this methodology, a total of five thermal fractions were collected for each sample. Briefly, a sample is heated at a constant rate (12°C min^−1^) from 40°C to 900°C under carrier gas flow composed of 75% N_2_ and 25% O_2_ (650 ml min^−1^ total). Any C released from the sample is fully oxidized to CO_2_ by a platinum catalyst held at 800°C. The produced CO_2_ is then quantified by a non-dispersive infrared (NDIR) detector. Carrier gas and CO_2_ then flow through a manifold consisting of parallel glass U-traps submerged in liquid nitrogen (LN_2_) under vacuum (10^−2^ mBar) and CO_2_ is cryogenically frozen and removed from the carrier gas. Once the sample reaches a desired upper-limit temperature, the first trap is sealed to trap CO_2_ produced at this temperature interval before a second parallel trap is opened and the next aliquot of CO_2_ is collected. The process is repeated for each subsequent temperature range.

The CO_2_ trapped at LN_2_ temperatures (−190°C) is then purified and quantified on a vacuum line using an isopropanol and dry ice trap to remove water and additional LN_2_ traps. A small CO_2_ subsample is collected for ^13^C measurement with a syringe and transferred to an He-flushed vial. The remainder is frozen into borosilicate glass tubes containing Ag and CuO wire and flame-sealed. Sample tubes are baked at 525°C for 1 h, during which the Ag and CuO remove additional contaminant gases (mostly N oxides that form from the reaction of N_2_ and O_2_ in carrier gases at high temperatures that freeze in the LN_2_ trap), necessary to avoid interferences with sample graphitization before ^14^C analyses (see §2b(iv)).

#### (ii) Activation energy

Activation energy (*E*_a_) distributions were calculated using the ‘rampedpyrox’ Python package [33,63]. For this, thermograms are transformed using a time-temperature model to density distributions of *E*_a_. In this study, *E*_a_ distributions and means (*μ_E_*) are used to describe the average bond strength of OC released, and standard deviations (*σ_E_*) to describe the heterogeneity of the bonding environment, where greater *σ_E_* indicates more diverse types and strengths of bonds. While these values should not be compared with *E*_a_ metrics determined via other methods, they can be used to compare samples measured under the same conditions.

#### (iii) Analytical pyrolysis (py-GC/MS)

The molecular structure of SOM was studied by analytical pyrolysis (pyrolysis gas chromatography-mass spectrometry: py-GC/MS) using a double-shot micro-furnace pyrolyzer (model 2020i; Frontier Laboratories Ltd., Fukushima, Japan) attached to a GC/MS system (Agilent 6890N/5973MSD, Agilent Technologies, Santa Clara, CA, USA). First, a general overview of the sample pyrolyzate was obtained by direct pyrolysis of the samples at 400°C [64]. Then, a sequential multishot pyrolysis approach was performed using the same five temperatures as those used for the thermal fractionation described above.

Briefly, soil samples (15–25 mg, depending on OC content) were placed in stainless-steel capsules (Frontier Laboratories Ltd. Eco-Cup LF) and introduced in a pre-heated pyrolysis micro-furnace at the starting temperature for 1 min. For the direct pyrolysis, the furnace starting temperature was set at 400°C and the pyrolysis was performed once in single shot mode. For sequential pyrolysis analysis, the furnace was set at the lowest temperature for each fraction, before a sample was introduced into the pre-heated furnace and thermal desorption was performed for 1 min. The resulting gases were directly injected into the GC/MS system. The sample in processing was immediately moved to a cold area of the pyrolyzer while the furnace temperature increased to the next temperature and the sample was reintroduced to the pyrolyzer hot area for 1 min. Following this procedure, the treatment was repeated for each temperature increment using the same sample. Thus, in total and using aliquots, six chromatograms per sample were analysed; one for the direct pyrolysis at 400°C and five for the sequential analysis, corresponding to each of the selected temperatures.

The GC was equipped with a low polar-fused silica (5% phenyl-methylpolysiloxane) capillary column (Agilent J&W HP-5 ms UI), of 30 m × 250 µm × 0.25 µm film thickness. The carrier gas was He with constant flow at 1 ml min^−1^. The oven temperature was held at 50°C for 1 min, increased to 100°C at 30°C min^−1^, from 100°C to 300°C at 10°C min^−1^ and stabilized at 300°C for 10 min. Mass spectra were acquired at 70 eV ionizing energy. The compound assignment was achieved via single-ion monitoring for various homologous series, low-resolution mass spectrometry and by comparison with published and stored (NIST and Wiley libraries) data. The relative abundance of each pyrolysis product was calculated as a percentage of the chromatographic area of all identified compounds.

#### (iv) C isotope analysis

In order to quantify the ^14^C in each sample, collected CO_2_ (see 2b(i)) was graphitized following the method of Steinhof *et al*. [65] and measured on a MICADAS AMS system (Ion Plus, Switzerland). Data were corrected for blank C contribution as described by Stoner *et al*. [34], and are expressed as Fraction Modern ^14^C (Fm) [66]. For the reader, we have also expressed Fm as an equivalent mean OC age by fitting the ^14^C data for the year of sample collection to a one-pool model as described by Khomo *et al*. [56].

Analysis of *δ*^13^C was performed on an aliquot collected via syringe during purification (§2b(i)) using a modified gasbench inlet to a continuous flow isotope ratio mass spectrometer (IRMS) [67].

#### (v) Rock-Eval

To assess the relative degree of decomposition and thermal lability versus stability of MOM and implied biogeochemical stability in soil [68], we applied Rock-Eval 6 pyrolysis analysis. In addition to commonly reported OI and HI values representing the ratio of H:C and O:C in SOM [69], we calculated I- and R-indices (‘immature’ and ‘refractory’, respectively) designed for SOM comparison [70].

Briefly, approximately 60 mg of powder-ground sample was added to a Rock-Eval 6 Turbo (Vinci Technologies, France, analysed by GEO-Data mbH, Garbsen, Germany) and underwent two consecutive heating phases, first in a pyrolysis oven (200–650°C; thermal ramping rate of 25°C min^−1^; under N_2_ atmosphere) then in a combustion oven (300–850°C; thermal ramping rate of 20°C min^−1^; under laboratory air atmosphere). At the beginning of pyrolysis, samples underwent an isothermal step at 300°C for 180 s, during which the free hydrocarbons (HC) were vaporized (S1 peak), before proceeding to higher temperatures as described above. The pyrolysis effluents (mostly HC) were detected and quantified with flame ionization detection, while CO and CO_2_ were quantified by infrared detection during both the pyrolysis and oxidation stages.

Rock-Eval 6 indices I (immature) and R (refractory) that better describe SOM were developed by Sebag *et al*. [70]. These I and R indices were calculated by comparing the relative areas of the pyrograms. Briefly, the I-index is equal to

$${\text{log}}_{10}\left(\frac{F1+F2}{F3}\right),$$

where *F*1, *F*2 and *F*3 are the relative areas of the deconvolution of Gaussian curves composing the S2 pyrogram. I-index values of SOM range from +0.64 in organic horizons to −1.32 in Bh horizons, although interquartile range is 0.11–0.34 for B horizons [70]. The R-index is defined as the proportion of the S2 pyrogram integrated after 400°C (a range of increasing refractory nature from 0.0 to 1.0). Note that low R-index values hereby indicate a low degree of thermal stability of SOM. By contrast, low I-index values indicate a high degree of SOM maturity and decomposition.

#### (vi) Caveats concerning pyrolysis versus oxidation

Thermal analysis techniques commonly measure soil characteristics with or without the presence of oxygen as an oxidizing agent, depending on the theory and goals of the research [71]. In this study, we employ both oxidative (thermal fractionation) and pyrolytic (py-GC/MS, Rock-Eval 6) methods to describe MOM characteristics with regard to increasing *E*_a_. The primary advantage of oxidation in our study is in minimizing artefacts due to charring, or the conversion of OC released at low temperatures to high-stability molecules rather than being released as a CO_X_ gas, potentially misrepresenting SOM thermal stability. Although the mechanisms of decomposition can vary between methods, previous studies have observed no significant difference in the *E*_a_ or the ^14^C measured in thermal fractions collected under ramped pyrolysis and oxidation [35,72]. Thus, we are confident that comparisons can be drawn between OC released along *E*_a_ gradients between both methods.

## 3. Results

### (a) Thermograms

**Figure 1.**
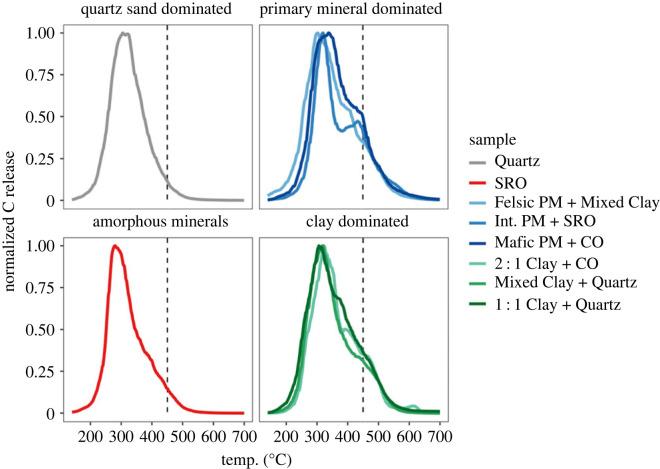
Thermograms of OC release as a function of temperature under oxidative conditions, heated at 12°C min^−1^. All thermograms are normalized by setting the peak of C release to a value of 1 in order to better compare soils with varying OC contents. Thermograms are grouped by dominant mineralogy (amorphous minerals, primary minerals, clays). The vertical dashed line indicates 450°C, above which OC is quantified by the index *T*_450–550_ to describe the high-temperature OC which may be strongly bound to clay minerals (see text).

Ramped thermal oxidation yielded distinct trends and differences between soils according to their dominant minerals (figure 1). All samples released 99% of total OC below 600°C, confirming the absence of calcium carbonate. All samples showed peak OC release around 320°C except ‘SRO’ with a peak at 285°C. The release of OC from the low-reactivity sample ‘Quartz’ and the short-range order amorphous mineral-rich sample ‘SRO’ followed an approximately normal distribution, with most OC released close to the temperature of peak release. Primary mineral- and clay-dominated sample thermograms had ‘shoulders’ after the main peak, indicating a smaller portion of SOM released at higher temperatures than the bulk of SOM in a sample. ‘Int. PM + SRO’ showed a second smaller, but distinct peak at 420°C.

High-temperature releases (450–550°C) likely contain OC that may be strongly associated with clay minerals, which is described by a thermal index (*T*_450–550_, figure 1 and table 2, [73]). In this temperature range (*T*_450–550_) there was significantly less (*t*-test, *p* < 0.001) high-temperature OC release in ‘Quartz’, and ‘SRO’ (2.8–4.2% total C, table 2) than the other samples (11.3–15.9% total C).

**Table 2.** Activation energy distribution data for all samples (sorted from lowest to highest *E*_a_), and slope of Fraction modern ^14^C (Fm) as a function of increasing *E*_a_ (kJ mol^−1^; figure 2). *T*_450–550_ is the percentage of total OC released between 450 and 550°C. ‘E peak’ is the *E*_a_ value at which the greatest magnitude of C release is observed.

dominant mineralogy	sample ID	*E* mean (*μ_E_*) (kJ mol^−1^)	*E* peak (kJ mol^−1^)	*E* std (*σ_E_*) (kJ mol^−1^)	youngest fraction (years)	oldest fraction (years)	slope Fm/*E*_a_ (kJ mol^−1^)	C release at *T*_450–550_ (%)
amorphous minerals	SRO	138.8	126.3	14.3	3582	4046	−0.0009	4.2
quartz sand	Quartz	139.4	135.1	13.4	845	1268	−0.0012	2.8
primary minerals	Mafic PM + CO	144.6	136.0	18.0	368	1404	−0.0022	11.3
clay	1 : 1 Clay + Quartz	146.1	134.2	17.5	584	1623	−0.0023	11.7
clay	Mixed Clay + Quartz	146.2	136.0	17.4	211	919	−0.0023	12.1
clay	2 : 1 Clay + CO	148.9	140.4	17.2	522	1713	−0.0027	12.9
primary minerals	Felsic PM + Mixed Clay	150.4	142.1	18.8	773	1478	−0.0014	12.9
primary minerals	Int. PM + SRO	152.1	136.0	19.1	766	1676	−0.0018	15.9

### (b) Activation energy

Similar grouping trends were observed in *E*_a_, as ‘SRO’ and ‘Quartz’ yielded significantly lower mean activation energies (*μ_E_*) than other samples (138.8–139.4 kJ mol^−1^; *t*-test, *p* < 0.01; table 2). Standard deviation of *E*_a_ distribution (*σ_E_*), a proxy measure for the diversity of OC bonding strengths, was also much lower in these samples (13.0–14.3 kJ mol^−1^; *t*-test, *p* < 0.01; table 2). Here, the three primary mineral-dominated soils had the highest *σ_E_* (‘Mafic PM + CO’ < ‘Felsic PM + Mixed Clay’ < ‘Int. PM + SRO’), suggesting the most heterogeneous bonds. Samples containing expandable 2 : 1 minerals and variable abundances of SRO and CO (‘2 : 1 Clay + CO’, ‘Felsic PM + Mixed Clay’, ‘Int. PM + SRO’) had significantly higher *μ_E_* (148.9–152.1 kJ mol^−1^) and high *σ_E_* (17.2–19.1 kJ mol^−1^).

### (c) Radiocarbon

Bulk soil mean ^14^C data (table 1) ranged from a low of 0.674 fraction modern (Fm) (one-pool model mean age of approx. 3200 years) measured for ‘SRO’ to a high of 1.009 Fm (most OC fixed in the last approx. 100 years) measured for ‘Mixed Clay + Quartz’. Most of the other soils had Fm ^14^C values between 0.85 and 0.99, indicating bulk mean ages of about 100–1500 years.

**Figure 2.**
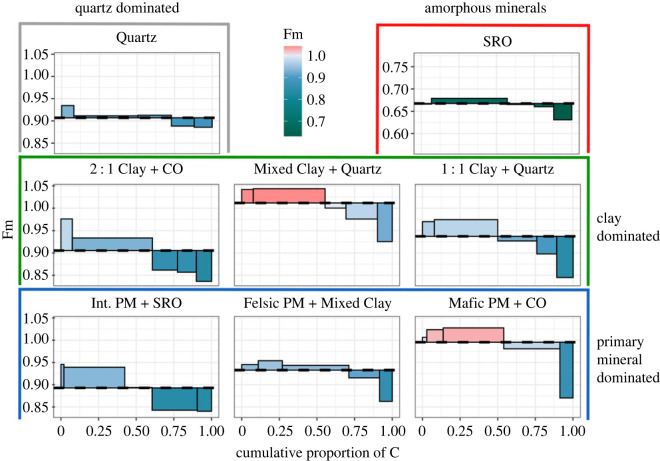
Fm ^14^C content for thermal fractions, represented as the proportion of total OC on the *x*-axis. Positive or negative height of bars indicates difference from the mean value (dashed horizontal lines). Fractions are ordered from low to high temperature. Note that the *y*-axis is absolute Fm value, and *y*-axis ranges are consistent between plots to show relative differences from mean values.

Despite the large range in mean MOM ^14^C, the difference in ^14^C between the thermal fractions and respective MOM values varied consistently, with the highest Fm ^14^C (youngest C) released at the lowest temperatures, and the lowest Fm ^14^C (oldest C) released at highest temperatures (figure 2 and table 2). For ‘SRO’ and ‘Quartz’, the samples with narrow thermograms with peak OC release at relatively low temperatures and thus the presumed weakest thermal stabilization mechanisms, the range in ^14^C age across thermal fractions was equivalent to a mean fraction age difference (oldest to youngest) of 420–460 years (table 2). By contrast, the mean ^14^C age difference between the oldest and youngest thermal fractions in samples with mixed mineralogies was larger, equivalent to a mean age difference of 700–1 200 years between OC released at the lowest and highest temperatures. In general, ^14^C content of thermal fractions decreased with increased *E*_a_ (figure 2 and table 2). However, there were distinct differences in the rate of decrease. ‘SRO’, ‘Quartz’ and ‘Felsic PM + Mixed Clay’ (in order) decreased in Fm with *E*_a_ much less rapidly (−0.0009 to −0.0014 Fm per kJ mol^−1^) than all other samples (−0.0018 to −0.0027 Fm per kJ mol^−1^).

### (d) py-GC/MS

**Figure 3.**
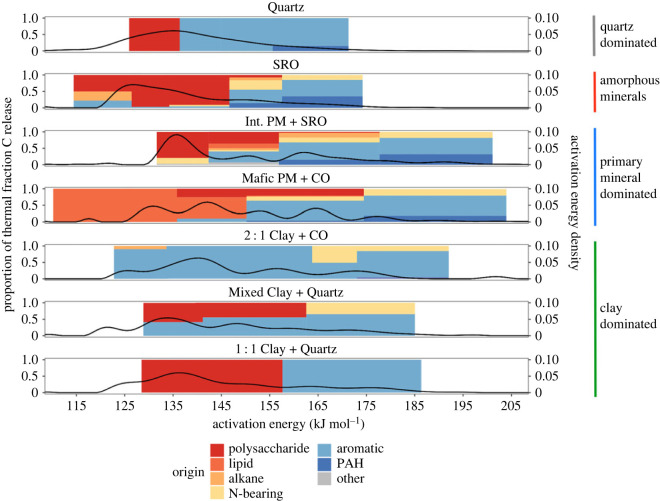
py-GC/MS profiles with calculated *E*_a_ probability distribution (PDF) functions calculated from oxidative thermograms overlain (black lines) to show the relative amount of OC associated with a given set of chemistry range of activation energies. PAH refers to ‘polycyclic aromatic hydrocarbons'. Right-hand axis represents density of OC released at a given temperature. Coloured bars on the right side correspond to sample groupings. ‘Felsic PM + Mixed Clay’ was not analysed.

Pyrolysis gas chromatography and mass spectrometry (py-GC/MS) revealed divergent trends in SOM quality and composition across mineralogies and temperatures (figure 3, electronic supplementary material, figures S4–S12). Of the 222 compounds detected across all samples, 74 were aromatic in nature, and 48 were polycyclic aromatic hydrocarbons (PAH). No lignin biomarkers (hydroxyphenyl, guaiacyl or syringyl subunits) were identified in any sample. Notably, the highest variety of organic compounds was detected in soil samples that contain various forms of SRO and CO. From those, the presence of SRO minerals had the strongest effect on chemical diversity, with 127 unique components detected in ‘SRO’, 85 in ‘Int. PM + SRO’ and 58 in ‘Mafic PM + CO’. Other samples contained fewer distinct compounds: in order, 14 in ‘2 : 1 Clay + CO’, 10 in ‘Quartz’ and 7 in both ‘1 : 1 Clay + Quartz’ and ‘Mixed Clay + Quartz’. Note that the sample ‘Felsic PM + Mixed Clay’ was not analysed due to late addition to the study and COVID-19 laboratory staff access restrictions.

With the exception of the ‘SRO’ sample, aromatic molecules were the most prominent group in all soils. Nitrogen-bearing compounds were detected in the ‘SRO’ soil, and soils with expandable 2 : 1 clay minerals. These compounds were generally released in thermal fractions of approximately 150 kJ mol^−1^ or more, but the two SRO soils (Int. PM + SRO and SRO) released N-containing OM with lower *E*_a_ than non-SRO soils (132–150 kJ mol^−1^). Polysaccharides, bioavailable compounds of potentially microbial origin, were detected in all samples except for ‘2 : 1 Clay + CO’. No soil released polysaccharides above approximately 178 kJ mol^−1^. Alkane/alkene chains were detected at low temperatures in ‘2 : 1 Clay + CO’ and ‘SRO’, while ‘Int. PM + SRO’ and ‘Mafic PM + CO’ (primary mineral-dominated soils) released alkanes with *E*_a_ > 175 kJ mol^−1^. The only strong lipid signals were detected in ‘Mafic PM + CO’ and a small amount in ‘Int. PM + SRO’, all below *E*_a_ of 178 kJ mol^−1^.

### (e) Rock-Eval soil organic matter indices

Rock-Eval SOM indices for all samples were in the expected range for B horizons [58] and followed the general trend of decreasing I-index values associated with increasing R-index values, suggesting a higher degree of SOM decomposition and more refractory SOM, respectively. Clay-dominated soils (‘2 : 1 Clay + CO’, ‘Mixed Clay + Quartz’, ‘1 : 1 Clay + Quartz’) showed the least thermally stable (low R-index) C, that was at the same time least decomposed (high I-index). Among the clay-dominated samples, ‘2 : 1 Clay + CO’, with high content of expandable clays, had thermally stable SOM (high R-index) that was more mature (low I-index). Interestingly, primary mineral-dominated soils (‘Int. PM, + SRO’, ‘Felsic PM + Mixed Clay’, ‘Mafic PM + CO’) had more stable SOM (high R-index) that was at the same time more decomposed (low I-index) than the clay-dominated soils. Overall, the highest R-index values were observed for the amorphous mineral-dominated soil ‘SRO’. Sample ‘Quartz’ was nearly centred for both indices and distinct from all other groups of samples.

## 4. Discussion

### (a) Mineral and organic controls on soil organic matter thermal stability, age distribution and chemistry

Our data confirm that the abundance, biochemical characteristics and mean ^14^C values of MOM can differ greatly depending on mineralogy and degree of mineral weathering. However, despite a variety of soil settings in our samples, we find distinct relationships between mineral reactivity, patterns of ^14^C distribution, and the type and number of chemical species in MOM. Following the hypothesis that greater activation energy (*E*_a_) corresponds to stability in soil, ‘labile’ forms of OC should be oxidized at lower temperature, decompose more easily, and be younger than the mean MOM ^14^C age, while high *E*_a_ OC should be older and more aromatic in nature [22]. This was generally observed across all samples (figure 3), with the additional observation that the low *E*_a_ OC was associated with pedogenic oxide minerals (SRO and CO), while high *E*_a_ OC was associated with clay minerals, especially 2 : 1 clays. Thus, fractionating MOM along a continuous *E*_a_ gradient allows us to describe which minerals tend to be associated with faster cycling (younger) or slower cycling (older) OC. This helps define SOM model pool structures and may have broad implications for assessing patterns of OC persistence across soil systems.

MOM forms through numerous types of interactions [4]. These include strong interactions between reactive functional groups in dissolved OM with reactive mineral surfaces, through weaker interactions between hydrophobic moieties in solution with surface-sorbed OM (e.g. hydrophobic seclusion [21,74]), or through the incorporation of local microbial necromass and exudates [75–78]. The thermogram and age structure of C oxidized thus reflects the overall stability of an organic moiety in a specific setting. Below, we briefly discuss the different types of mineral–OM interactions analysed (e.g. SRO, CO, clays and primary minerals) and patterns of OC chemistry and relative age distributions associated with each. As most soils, even ones predominantly consisting of clay minerals, can have substantial amounts of OC associated with pedogenic oxides and oxyhydroxides, we also discuss patterns observed in mixed mineralogy soils.

#### (i) Amorphous mineral (soil organic matter) rich samples

**Figure 4.**
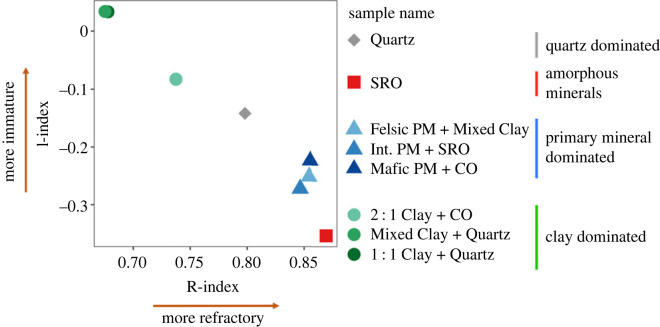
Results of Rock-Eval 6 SOM across samples calculated to assess the degree of decomposition using I-index (immaturity) and the thermal stability using the R-index (refractory) [70].

Reactive SRO minerals display the capacity to store large amounts of OC over long time spans and can protect a diverse range of compounds from mineralization by creating energetic or physical barriers that deter microbial decomposers [2,79,80]. Despite the majority (main thermogram peak) of the MOM in ‘SRO’ being oxidized at relatively low *E*_a_ in mostly bioavailable forms, it contained very old OC (Fm 0.63–0.67, figures 2 and 3, table 1). The narrow *E*_a_ range and ^14^C distribution of MOM in ‘SRO’ (table 2) indicate homogeneous stabilization mechanisms, such as OM–OM bonds or co-precipitation of metals and OM [81,82], that do not provide strong protection under thermally *oxidizing* laboratory conditions. However, as indicated by the overall age of the bulk MOM and high *pyrolytic* Rock-Eval R-index (figure 4), the thermal oxidation method may bypass physical protection mechanisms that can be responsible for greater mean OC ages. This interpretation is supported by the presence of more diverse organic compounds in soils with expandable clays as well as amorphous SRO minerals, e.g. ‘Int. PM + SRO’ (figure 3).

#### (ii) Quartz sample

In Podzol B horizons, pedogenic Al and Fe oxides are the primary method of OC stabilization. The ‘Quartz’ sample in this study was composed of wind-blown quartz sand (100% sand by texture, [54]), with only small amounts of extractable Fe (table 1). Limited stabilization pathways resulted in the narrow thermogram and ^14^C distribution (figures 1 and 2). The simplistic chemistry reflects stronger, aromatic accumulation in hydrophobic fractions via ligand exchange (higher temperatures), and weaker, bioavailable polysaccharides in the hydrophilic fractions (lower temperatures) [83,84], although the high sand content may have favoured carbohydrate accumulation [85]. We can attribute the long transit times (845–1268 years, table 2) in this subsoil to low OC and nutrient concentrations limiting microbial decomposition [86,87].

#### (iii) Clay mineral-rich samples

Compared to clay-poor samples ‘SRO’ and ‘Quartz’, clay minerals, even in low abundance, increase the range of activation energies and ages (table 2) detected within a sample as observed most strongly in ‘2 : 1 Clay + CO’, the most clay-rich soil analysed (47% clay by texture, 93% of which is smectite, table 1). Clay content, as well as total N content, has been found to correlate with thermal stability, with direct mineral–OC associations attributed to C release in the temperature range of 450–550°C ([73,88,89]; figure 1). The 200–300°C temperature range has previously been attributed to bioavailable SOM components, as the amount of C released at low *E*_a_ decreased the most after soil incubation [90,91]. In our samples, most OC in clay-dominated soils is released between 200 and 400°C, and is thus likely composed of OC released from organo–organo bonds and organo-mineral bonds with co-occurring (oxy)hydroxides [92–94] that may precipitate on clay surfaces [29,55], with older, clay edge-bound OC releasing at higher temperatures [73,95]. Notably, among the clay-dominated and primary mineral-dominated soils, we did not observe strong effects of clay type (1 : 1 versus 2 : 1) on thermal stability (figure 1) despite evidence that different clays sorb very different SOM ([3,89,96,97]; figure 3). The predominance of polysaccharides released at low temperature, and aromatics and N-bearing SOM released at high temperature, respectively, suggests two distinct pools of SOM with different pathways of clay-mineral stabilization: (i) younger, bioavailable polysaccharides associated with greater microbial activity (root exudation and greater microbial enzyme activity [96,98]) on clays [99] and (ii) dominantly aromatic OC bound to clays via hydrophobic exclusion, cation bridging and hydrogen [3,4].

#### (iv) Organic matter quality patterns

Polysaccharides make up the majority of the main peak of OC release in most soils (figure 3) and showed mean ages of approximately 200 up to approximately 1 000 years (albeit approx. 3 400 years for ‘SRO’) (table 2 and electronic supplementary material, table S1). However, N-bearing compounds are a critical MOM pool that can persist on minerals for centuries, despite N limitation in most soils (figure 2, [100–104]). Generally, proteins and amino sugars are strongly amphiphilic [105] and bear one of the few SOM functional groups (–NH_X_) that may be positively charged, thus forming strong bonds with negatively charged mineral surfaces, e.g. oxides and 2 : 1 clay minerals [106]. Even more importantly, they may act as the first ‘wetting’ layer upon which subsequent OM can bind, although potentially more weakly [106–109]. Indeed, we observed that only soils containing smectite (‘2 : 1 Clay + CO’, ‘Mixed Clay + Quartz’, ‘Int. PM + SRO’ and ‘Mafic PM + CO’) and SRO minerals (‘SRO’ and ‘Int. PM + SRO’) yielded N-containing C, released at high *E*_a_ and in the oldest thermal fraction within each sample (figure 3). Following a multi-layer model of MOM accumulation, the outer layers of MOM may be oxidized at lower *E*_a_, giving way to N-bearing and aromatic OC bound to mineral surfaces. However, the exact processes of MOM oxidation that can disrupt OM–OM as well as OM-mineral bonds are still unknown [109]. In the case of allophane soils (SRO), it is not clear if these N-bearing compounds are preserved preferentially or included in the diverse MOM in sample ‘SRO’ that has at the same time a low immaturity index and high ^14^C age (figures 2 and 4, table 2). We argue that if this OC is of microbial origin, as evidenced by the lack of lignin biomarkers (figure 3), it indicates active cycling of rather old C, highlighting the importance of considering pathways for pre-aged OC to enter MOM when interpreting these ^14^C values.

In summary, soils that were able to provide better protection for SOM (older ^14^C age and higher mean *E*_a_) were also able to stabilize bioavailable OC species against decomposition (figure 3). While in general, our hypothesis held that greater *E*_a_ was associated with older and more aromatic OC within a given soil, the overall bulk ^14^C did not follow the expected patterns. The overall oldest OC was in the ‘SRO’ soil, yet it released OC with lower *E*_a_ than OC associated with clay in other soils, which was younger (figures 1 and 2). Furthermore, the high R-index (figure 4, electronic supplementary material, table S2) under pyrolytic Rock-Eval analysis, during which allophane may degrade to more crystalline minerals at temperatures greater than 600°C [110], likely provides a better indicator of the apparent long-term stability of MOM in ‘SRO’ than oxidative thermograms alone. Thus, the pedogenic setting of samples plays the dominant role in determining the absolute age of OC in B horizons, while mineralogy controls how OC age is distributed around the mean ^14^C value.

### (b) Mineral-associated organic matter as a heterogeneous ^14^C pool

#### (i) Implications for pool model structure

To explain observations that include the response of SOM over time to changes in vegetation inputs, or to fit time series of ^14^C over the last decades has required models to represent SOM using pools with different timescales of turnover, ranging from years to millennia. Thermal analysis approaches help determine which mineral stabilization mechanisms may give rise to different model structures. For example, in subsoils with mixed mineralogy (‘2 : 1 Clay + CO’, ‘Mixed Clay + Quartz’, ‘1 : 1 Clay + Quartz’, ‘Int. PM + SRO’, ‘Felsic PM + Mixed Clay’, ‘Mafic PM + CO’, table 1) our results suggest that faster cycling SOM is more bioavailable and potentially associated with more crystalline metal oxides and/or weaker OM–OM bonds, while older MOM is associated either with amorphous SRO (reflecting potentially occlusion or physical isolation) or expandable 2 : 1 clay minerals that have a larger reactive surface area and strong ionic stabilization of OC (figures 2 and 3). By contrast, SOM turnover in soils dominated by a single mineral (e.g. ‘Quartz’, ‘SRO’) could potentially be modelled as a single pool. Indeed, thermal fractionation indicates that the range of ^14^C ages within a given thermogram feature can be relatively narrow (figure 2), indicating that mixed mineral soils with clay can be modelled as two homogeneous pools with different mean C ages. These pools also reflect different chemical composition, with low-temperature OC reflecting microbial polysaccharides while OC released at higher temperatures consists of slowly cycling aromatic and N-bearing OC (figure 3).

#### (ii) Linking age distributions and model structures

Isolating fractions of varying thermal stability and *E*_a_ in combination with describing the chemical quality of MOM from distinct thermal fractions proved to be a useful tool for separating SOM according to the type of mineral with which it is associated and its relative age in distinct soils. While the directional changes of processes in complex soil systems are not well understood and are difficult to predict [89], thermal fractionation may have the potential to identify the proportion of the MOM in subsoils that is more weakly stabilized and thus may be more vulnerable to change on multi-decadal to multi-centennial timescales. We found that MOM contains molecules with diverse chemistry, *E*_a_, and age dependent on mineral composition (figures 2 and 3). More research is needed to understand the evolution of soil age/depth distributions using gradients that can track changes in minerals and OC age through time across soil of varying development trajectories. Such studies are also needed to understand the potential vulnerability of different mineral–OC associations to climatic or vegetation change.

#### (iii) Controls on absolute organic carbon age

While thermal oxidation clearly provided a distribution of ^14^C ages within a given sample, we were not able to predict the mean ^14^C age of a sample from mineralogy or OM chemistry alone. For example, polysaccharides released at low *E*_a_ (<160 kJ mol^−1^) make up the majority of OC release in most soils (figure 3) but had mean ages ranging from approximately 200 up to approximately 1000 years (and approx. 3400 years for ‘SRO’, table 2 and electronic supplementary material, table S1). The mean age of C can reflect a number of processes. For example, the pre-ageing of OC in roots or plant stems, or in recycling of older C released from minerals by microbes can provide ‘fresh’ substrates that were originally fixed from the atmosphere hundreds of years previously. Another mechanism for pre-ageing of C inputs at depth reflects rates of vertical transport from regions of high C input to lower C availability. For example, the ^14^C content of SOM arriving in subsoil (e.g. Spodosol B horizons) reflects the mean time required for OC to arrive in that horizon and is not necessarily a good measure for the *in situ* stability or decomposition rate of B horizon SOM [111]. Thus, the age of OC in a given pool may not reflect its present vulnerability to destabilization or decomposition.

Future climates may de-stabilize OC through enhanced microbial activity [51,112], enhanced weathering [7,80,113,114] and changing redox conditions [80,115,116]. Combining the effects of environmental, site-specific conditions with an improved understanding of the dynamics of soil C stabilization dynamics and mineral stability may better constrain transit times of SOM and greatly improve the predictive power of soil C turnover models. For example, a large fraction of OC in our mixed mineralogy soils is associated with pedogenic oxides but situated in greatly different bioclimatic settings. Future studies could test the degree to which the age and age distribution of associated OM relates to the stability of minerals or the conditions that control C input and turnover by studying gradients of redox cycling or changes in pH associated with root exudates or microbial enzyme activities. Moving forward, MOM must be considered a large, dynamic pool of OC that is not insensitive to changing environmental conditions.

## 5. Conclusion and future work

A direct comparison of diverse MOM using a variety of thermal oxidation and pyrolysis methods successfully highlighted the distinct effects of soil minerals on MOM persistence. Soils with singular mineral stabilization pathways and without clay minerals had narrower ^14^C and energy distributions and, excluding soils rich in amorphous SRO minerals, stored generally less chemically diverse compounds with younger ^14^C ages. Soils containing a larger variety of minerals offered multiple stabilization mechanisms leading to a much wider range of activation energies, SOM ages, and generally greater diversity of organic compounds. Through the combination of several thermal stability analyses and characterization of MOM molecular structure, we were able to identify distinct timescales for MOM turnover and the biochemical structure of MOM associated with different types of soil minerals. MOM in soils containing 2 : 1 clays had overall higher thermal stability and released older OC at higher temperatures than soils dominated by non-expandable 1 : 1 clay minerals or highly crystalline minerals. In all soils, the majority of MOM appears to be associated with (pedogenic) (oxy)hydroxides and amorphous SRO minerals or weakly stabilizing OM–OM bonds. These minerals were able to store abundant and diverse OC compounds, including potentially bioavailable and more accessible SOM. Thus, our results support previous studies showing the role of oxides as dominant drivers of total SOM storage, but also show a smaller but significant role of clays in strongly stabilizing relatively old and chemically distinct C. Thermal oxidation demonstrated that it can successfully distinguish OC associated with different minerals and provide predictable timescales and chemical characteristics. We conclude that while the directional changes of processes in complex soil systems are not well understood and remain difficult to predict, thermal fractionation may have the potential to identify SOM that is more weakly stabilized and more likely to be vulnerable to future change on multi-decadal to multi-centennial timescales. The mineralogical control on the age structure of MOM has important implications for how soil SOM models need to account for differences in soil mineralogy, and how specific portions of SOM may have distinct reactions to changing environmental conditions that affect plant C input, microbial C turnover and mineral OC stabilization to varying degrees and at varying timescales.

## Data Availability

Data and code will be maintained in this repository: https://github.com/ShaneStoner/Mineralogy14C. It is also available with a DOI via Zenodo: https://zenodo.org/record/7998659 [117]. The data are provided in electronic supplementary material [118].
